# Supplementary material for: Availability, price and affordability of insulin, delivery devices and self-monitoring blood glucose devices in Indonesia
Source: PLoS One. 2024 Oct 3;19(10):e0309350. doi: 10.1371/journal.pone.0309350 (PMC11449349; doi:10.1371/journal.pone.0309350)
Supplement: Attachment — Submitted filename: Response to Reviewers.docx [file pone.0309350.s002.docx]

**PONE-D-24-04996**

**Availability, price and affordability of insulin, delivery devices and self-monitoring blood glucose devices in Indonesia**

Dear Academic Editor and Reviewers,

We sincerely express our gratitude for your insightful and critical comments on our manuscript. We have modified the manuscript in response to your remarks. We do hope that the revised manuscript will be considered by PLOS ONE. We will respond to the comments point counter point.

-----------------------------------------

**Journal Requirements:**

1. When submitting your revision, we need you to address these additional requirements. Please ensure that your manuscript meets PLOS ONE's style requirements, including those for file naming.

**RESPONSE: we have prepared the revised version and its supporting documents, so they are in line with PLOS ONE’s style requirements.**

2. We suggest you thoroughly copyedit your manuscript for language usage, spelling, and grammar. If you do not know anyone who can help you do this, you may wish to consider employing a professional scientific editing service.

Upon resubmission, please provide the following: The name of the colleague or the details of the professional service that edited your manuscript

**RESPONSE: we have not used professional editing services. Three of our co-authors are native English-speakers so they assisted by editing the revised manuscript.**

3. We note that the grant information you provided in the ‘Funding Information’ and ‘Financial Disclosure’ sections do not match.

When you resubmit, please ensure that you provide the correct grant numbers for the awards you received for your study in the ‘Funding Information’ section.

**RESPONSE: we have provided the ‘Funding Information’ including the grant number. We ensure that the ‘Funding Information’ matches with ‘Financial Disclosure’.**

4. We note that your Data Availability Statement is currently as follows: [All relevant data are within the manuscript and its Supporting Information files]

Please confirm at this time whether or not your submission contains all raw data required to replicate the results of your study. Authors must share the “minimal data set” for their submission. PLOS defines the minimal data set to consist of the data required to replicate all study findings reported in the article, as well as related metadata and methods (<https://journals.plos.org/plosone/s/data-availability#loc-minimal-data-set-definition>).

Authors do not need to submit their entire data set if only a portion of the data was used in the reported study.

**RESPONSE: The submission includes the aggregated data (minimal data set). The submission does not include facility-level data as the names of the facilities are confidential.**

5. Please include a copy of Table 4 which you refer to in your text on page 12.

**RESPONSE: we have checked our manuscript and realized that we made an error. There is no Table 4. It should have been written as Table 2 which we have corrected in the revised manuscript.**

**Additional Editor Comments:**

Authors need to revise the methodology based on the reviewer comments. Authors also need to revise the discussion and the conclusion based on the aim of this study. They need to explain more detail about the findings.


[Note: HTML markup is below. Please do not edit.]

**RESPONSE: We have made some changes on Methods, Discussion and Conclusion based on comments from reviewers. The changes can be seen in the manuscript now revised with track changes.**

**Reviewers' comments:**

Reviewer's Responses to Questions

**Comments to the Author**

**Reviewer #1**

1. This is a very interesting study with important findings. However, the study does not exactly follow WHO/HAI methodology while doing pricing calculations. This needs to be described clearly in the methods/discussion and limitations sections of the study.

   Kindly mention the following

   The study does not exactly follow a WHO/HAI methodology but a variation and the prices are reported in USD (instead of a median price ratio)

   This could be partly because of the absence of an updated international MSH drug pricing indicator guide (as the last one was published in 2015)

**RESPONSE: Thank you for your suggestion. We did not report price ratios as we felt no suitable external reference price (including one that lists prices for medical devices) was available. We did not report in Indonesian Rupiah as Plos One is a global journal.**

**We have modified the Methods by providing information on the variation (i.e. price reporting in US$ instead of median price ratios or prices in local currency) that we made from the original version of WHO/HAI methodology (manuscript with track changes line 28-29, 177-178). We have also acknowledged the variation as the part of limitations of this study (manuscript with track changes line 536-538).**

2. Also at places, sentences and paragraphs need support references (for example below)

Thirdly, the lack of a universally accepted definition of affordability may impede the accurate comparison of our findings with those of other studies.

**RESPONSE: we have provided the references to support some sentences (Manuscript with track changes line 534,536)
We agree the lack of a universally accepted definition of affordability is a challenge. The co-authors from HAI have lobbied WHO to develop a definition which we have been told is in process.**

**Reviewer #2**

1. Line 176: The authors have used 150 test strips. Is this for a month or the entire two years? If this is meant for a month, I would suggest to use the Indonesian guidelines. Indonesia, being a middle-income country, it is less likely for a diabetic to do 6-8 self-assessment per day.

**RESPONSE: 150 test strips are used for a month’s supply. If we refer to the Indonesian Guidelines for Diabetes Management published by Indonesian Association of Endocrinologist in 2021, the number of tests per day is not explicitly stated. However, the association recommends self-testing several times a day for uncontrolled patients including before each meal, 2 hours after each meal and before bedtime prior meals. The frequency of self-testing recommended in the Indonesian guideline is similar with that of American Diabetes Association. This is now stated in the revised manuscript.**

2. Line 203-204: “As human insulin was not available in public health facilities, it is unsurprising that no insulin syringe was provided in this sector.” I tend to defer with the authors as to the unavailability of insulin syringes in public facilities. This is because of certain types of analogue insuring requires syringes for administration. Please re-look at this.

**RESPONSE: Currently analogue insulins in the Indonesian market are available only as pre-filled pens. Analogues in cartridges or vials require syringes to inject into pumps, but neither cartridges or vials were found.**

3. Line 396-451. This section seems to be new and does not relate to the title and the flow of the rest of the document. If the authors need to include it, they need to either alter the title, or add another secondary objective of the study to match with the discussion

**RESPONSE: We do agree with the reviewers to modify our objectives (see manuscript with track changes line 138-139)**
